# Phenprocoumon based anticoagulation is an underestimated factor in the pathogenesis of calciphylaxis

**DOI:** 10.1186/s12882-019-1301-6

**Published:** 2019-04-02

**Authors:** Philipp Russ, Martin Russwurm, Birgit Kortus-Goetze, Joachim Hoyer, Sahana Kamalanabhaiah

**Affiliations:** Department of Nephrology, University Hospital of Marburg, UKGM GmbH, Baldingerstraße 1, 35033 Marburg, Germany

**Keywords:** Calciphylaxis, Inflammation, Ischemia, Vascular calcification, Phosphatemia

## Abstract

**Background:**

Calciphylaxis is a life threatening complication in renal patients. Of great importance is the identification of concomitant factors for calciphylaxis. Due to the variability of clinical presentation the evaluation of such factors may be obscured when calciphylaxis diagnosis is based just on clinical features. We aimed to characterize associated factors only in patients with calciphylaxis proven by histomorphological parameters in addition to clinical presentation.

**Methods:**

In a single center retrospective study we analyzed 15 patients in an 8 year period from 2008 to 2016. Only patients with clinical features and histomorphological proof of calciphylaxis were included. Criteria for histological diagnosis of calciphylaxis were intimal hyperplasia, micro thrombi or von Kossa stain positive media calcification.

**Results:**

The mean age of patients was 64.8 years. Nine patients (60%) were female; 12 (80%) were obese with a Body-Mass-Index (BMI) > 30 kg/m^2^; 3 (20%) had no renal disease; 12 (80%) had CKD 4 or 5 and 10 (66.7%) had end-stage renal disease (ESRD). One-year mortality in the entire cohort was 73.3%.

With respect to medication history, the majority of patients (*n* = 13 (86.7%)) received vitamin K antagonists (VKA); 10 (66.7%) were treated with vitamin D; 6 (40%) had oral calcium supplementation; 5 (33.3%) had been treated with corticosteroids; 12 (80%) were on proton pump inhibitors (PPI); 13 (86.7%) patients had a clinical proven hyperparathyroidism. Ten (66.7%) patients presented with hypoalbuminemia at diagnosis.

**Conclusions:**

The evaluation of biopsy proven calciphylaxis demonstrates that especially treatment with vitamin K antagonists and liver dysfunction are most important concomitant factors in development of calciphylaxis. As progression and development of calciphylaxis are chronic rather than acute processes, early use of DOACs instead of VKA might be beneficial and reduce the incidence of calciphylaxis.

## Background

Calciphylaxis, also known as calcific uremic arteriolopathy (CUA), is a rare disease associated with chronic kidney disease (CKD). Crystals precipitate in the media of small cutaneous blood vessels presumably because of an imbalance of serum calcium- and phosphate-levels [1] as well as a disorder of metabolic modulators such as parathyroid hormone [2]. The resulting perivascular calcification provokes painful vascular obliteration, ulceration and necrosis. The one-year-mortality ranges between 50 and 70% [1, 3] and is mainly due to infectious complications or sepsis. Patients suffering end-stage renal disease (ESRD) are predominantly affected by calciphylaxis, however, calciphylaxis also occurs in patients with moderately impaired or even normal renal function [4]. With an estimated incidence of <1% [5] amongst ESRD, prospective clinical trials are difficult to conduct due to the rarity of the disease.

Before the diagnosis of calciphylaxis can be made, several other entities like peripheral artery disease, vasculitis, phenprocoumon or heparin induced skin necrosis, cryoglobulinemia and cholesterol embolization syndrome need to be considered. Pathological laboratory findings of parathyroid hormone, serum calcium- and phosphate-levels are typical but not disease defining. Furthermore, secondary hyperparathyroidism and disturbed calcium and phosphate homeostasis are common co-morbidities in patients with ESRD. Gold standard for diagnosis of calciphylaxis is a positive skin biopsy with well defined diagnostic criteria like the von Kossa stain positive media calcification, intimal hyperplasia, micro thrombi, epidermal ulceration or extravascular soft tissue calcification [6]. However, in the majority of studies the rate of biopsy proven calciphylaxis is low and diagnosis is often facilitated by clinical and laboratory features [7]. On the other hand, the lack of biopsy probably leads to inclusion of ulceric lesions not due to calciphylaxis. Therefore, we performed a study of associated factors for calciphylaxis including only patient with biopsy proven calciphylaxis.

Our primary objective was to assess concomitant factors that might contribute to either outbreak or promote an aggravated course of calciphylaxis in order to develop a deeper understanding of the etiology of calciphylaxis. In order to do so, we collected data on laboratory parameters, epidemiology and the survival rates in patients suffering from biopsy proven calciphylaxis at the university hospital of Marburg (Germany). Furthermore, our focus was on the treatment of patients, examining factors influencing the calcium metabolism and calcification.

## Methods

We conducted a retrospective analysis of patients from University hospital of Marburg, searching for the International Classification of Diseases (ICD) code for calciphylaxis (E83.50) in the clinics documentary system “Orbis” (AGFA®). The recruitment period was 2005–2016. By this method we could identify 20 patients.

Only those patients with strong clinical evidence of calciphylaxis and with positive histological findings were included. A distinct histological finding for calciphylaxis includes the von Kossa stain positive media calcification or at least one of the secondary changes such as intimal hyperplasia, micro thrombi or soft tissue calcification [6]. Therefore, in accordance with dermatohistology all patients included showed a histology compatible with calciphylaxis. Patients with clinical evidence but without dermatohistological proof or lacking biopsy were excluded.

Data on epidemiology, co-morbidity, medication and treatment before diagnosis, laboratory parameters as well as the survival rate were taken from electronic patient record. Data analysis was done with descriptive statistics.

## Results

### Epidemiology and patients co-morbidities

All 15 Caucasian patients showed a biopsy proven calciphylaxis. Table 1 summarizes patient demographics and co-morbidities. At diagnosis mean age was 64.8 years (SD 15.4 years) with an age range of 20–80 years. Nine patients (60%) were female. No patient had a BMI < 25 kg/m^2^. According to the International classification of adult obesity, 3 patients were pre-obese (BMI 25.00–29.99), 3 were class one obese (BMI 30.00–34.99), 2 class two obese (BMI 35.00–39.99) and 7 patients were class three obese (BMI 40 or higher). Proximal body lesions were seen in the majority of cases (11, 73.3%), however, 4 (26.7%) patients only had distal lesions. Table 1 summarizes patient co-morbidities before diagnosis of calciphylaxis. Three patients (20%) had no kidney disease. 12 (80%) patients presented with pathological kidney laboratory parameter such as creatinine, urea, glomerular filtration rate and urinary parameters or showed a biopsy proven nephropathy. These patients could be classified to CKD stages 4–5. Before diagnosis 10 patients had ESRD, of which 3 had begun dialysis within the last 6 month. We measured PTH levels in all patients. Hyperparathyroidism was defined as an elevation above the upper limit of normal for the assay in use (> 65 ng/l). Thirteen patients (86.7%) showed a hyperparathyroidism (median PTH level = 157 ng/l; interquartile range = 73–241 ng/l). All cases of hyperparathyroidism were secondary in nature. Seven (46.7%) patients had concomitant type 2 diabetes and 2 (13.3%) a liver disease. One year mortality was 73.3%.

**Table 1:** Demographics and co-morbidities

Characteristic	Baseline, *n* = 15
Age, mean (SD)	64.8 (15.4)
Caucasian, n (%)	15 (100)
Sex, n (%)	
Male	6 (40)
Female	9 (60)
Obesity with BMI > 30 kg/m^2^, n (%)	12 (80)
Clinical manifestation of calciphylaxis	
Proximal body lesions, n (%)	11 (73.3)
Only distal body lesions, n (%)	4 (26.7)
CKD stage 4 and 5, n (%)	12 (80)
CKD5D, n (%)	10 (66.7)
Hyperparathyroidism, n (%)	13 (86.7)
Type 2 Diabetes, n (%)	7 (46.7)
Liver disease, n (%)	2 (13.3)
Biopsy proven calciphylaxis, n (%)	15 (100)
One-year mortality, n (%)	11 (73.3)

### Laboratory parameters at point of diagnosis

Table 2 summarizes laboratory parameters associated with disturbed homeostasis in patients with CKD and identified as potential concomitant factors for the development of calciphylaxis. The majority of patients (9 out of 15, 60%) showed an increased serum-phosphate level. 46.7% of all patients received phosphate binders. Although, 10 were on vitamin D supplementation and 6 patients received oral calcium, serum-calcium levels were low in 7 patients. Only one patient had increased serum calcium levels, all others (46.7%) had normal serum calcium levels. However, after correcting calcium for serum albumin levels, 7 patients (46.7%) showed hypercalcaemia (> 2.58 mmol/l). At the time of diagnosis 10 patients were hypoalbuminemic. During disease progression serum-albumin levels of 13 patients were decreased. Furthermore, pseudocholinesterase (PCHE) as a marker of liver synthesis was examined. Data was only available in 10 (66.7%) patients, but all PCHE serum levels were reduced. Alkaline phosphatase was elevated in 6 (40%) patients.

**Table 2:** Laboratory parameters in patients suffering calciphylaxis

Laboratory parameter at point of diagnosis	Baseline, *n* = 15
Serum-phosphate level	
Normal, n (%)	6 (40)
Elevated, n (%	9 (60)
Total serum-calcium level	
Normal, n (%)	7 (46.7)
Reduced, n (%)	7 (46.7)
Elevated, n (%)	1 (6.7)
Calcium corrected for albumin	
Normal, n (%)	8 (53.3)
Elevated, n (%)	7 (46.7)
Hypoalbuminemia, n (%)	10 (66.7)
Elevated alkaline phosphatase, n (%)	6 (40)

### Histologic findings

Mochel et al. [6] studied histologic features of calciphylaxis and included histological features such as microthrombi, intimal hyperplasia, epidermal ulceration and extravascular soft tissue calcification. All patients showed calciphylaxis defining histological features. Two thirds showed a von Kossa stain positive calcification of small and medium-sized skin vessels. One third showed no calcification but secondary histological changes associated with calciphylaxis such as intimal hyperplasia, microthrombi or extravascular soft tissue calcification (Table 3).

**Table 3:** Histologic findings in patients of our cohort

Histological findings	Baseline, *n* = 15
Bioptic result compatible with calciphylaxis	15 (100)
Von Kossa stain positive calcification	10 (66.7)
Secondary caused changes	5 (33.3)

### Medication at time of diagnosis

**Fig. 1:**
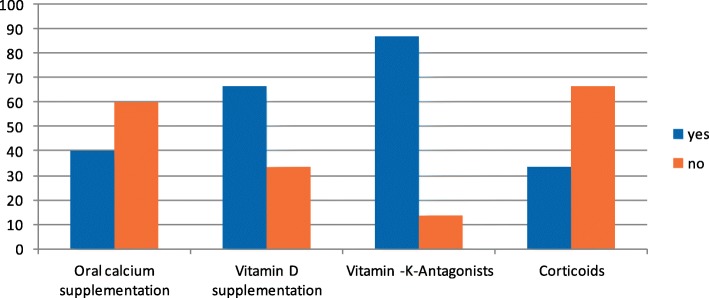
Medication at time of diagnosis

Table 4 summarizes specific medication. Figure 1 (medication at time of diagnosis) shows commonly observed concomitant medication in patients with calciphylaxis, as identified by various authors [8, 9]. 13 (86.7%) patients were on vitamin-K-antagonist due to atrial fibrillation. Two thirds (10, 66.7%) received vitamin D, 6 (40%) were treated with oral calcium supplementation. One third (5 patients, 33.3%) were on systemic corticosteroids.

**Table 4:** Medications in patients suffering bioptic proven calciphylaxis

Preexisting Medication at time of diagnosis	Baseline, *n* = 15
Vitamin K antagonists, n (%)	13 (86.7)
Vitamin D supplementation, n (%)	10 (66.7)
Oral calcium supplementation, n (%)	6 (40)
Corticosteroids, n (%)	5 (33.3)
PPI, n (%)	12 (80)
Ferritin supplementation, n (%)	5 (33.3)
Statin, n (%)	7 (46.7)

PPI are known to cause a statistically significant difference in serum total calcium and parathyroid hormone (PTH) [10] and thus medication was of interest. In our cohort, 12 (80%) patients were on PPIs.

Similarly, iron supplementation has been suspected of having a negative effect on calciphylaxis [11, 12]. In our cohort, 5 patients received intravenous ferritin (33.3%). Statins, in turn, have been associated with anticalcific, antithrombotic and anti-inflammatory properties in patients with calciphylaxis [13]. In our series 7 (46.7) patients received statins.

## Discussion

In this retrospective analysis, we could identify multiple interacting factors, which may be associated with the outbreak of calciphylaxis. There seems to be no single, specific pattern of factors that leads to this disease. However, we identified the factors that seemed to be the most common and clinically significant. Certainly, the individual factors also differ in their weight.

We can confirm that aside from secondary hyperparathyroidism, obesity and female gender seem to be major concomitant factors. Furthermore, treatment with VKA is an important factor associated with calciphylaxis, present in almost 90% of patients. Additionally, the majority of patients were treated with PPI.

Most patients suffered from secondary hyperparathyroidism due to CKD and consecutive disturbed calcium and phosphate metabolism, which in turn poses a factor associated with the outbreak of calciphylaxis, as many previous studies have shown [4, 14–16]. Nevertheless, only few patients showed elevated calcium -phosphate product. This may be due to therapeutic intervention in the calcium and phosphate metabolism through oral calcium and vitamin D supplementation as well as calcium containing phosphate binders. In any case, there is equal evidence for either supporting or disputing an effect of the calcium phosphate product on the development of calciphylaxis [13, 17–19].

This way, although a pathological calcium-phosphate product is commonly found in patients suffering from calciphylaxis, it is not a requisite for diagnosis and may be absent in some. Another factor influencing the calcium and phosphate homeostasis is the supplementation of oral vitamin D, which was found in the majority of our calciphylaxis patient before outbreak of the disease. This observation is substantiated by several studies and case reports [4, 9, 13, 17]. Similarly, PPI seem to influence serum magnesium and calcium levels, which in turn can lead to decreased calcium-levels [10]. Noticeably 80% of our patients were on PPI. To our knowledge there is no current biological rationale for a contribution of PPI to calciphylaxis and future studies exploring a possible pathophysiological connection might be of interest.

The most noticeable result of our study was the high percentage of treatment with VKA (almost 90%). Other studies estimate VKA usage at approximately 25% [4, 20]. VKA is an established concomitant factor for the development of calciphylaxis and accelerates the calcification process. Studies using VKA treated rat models not only show a more rapid calcification in arteries but also an effect on heart valves [21]. VKA inhibits the posttranslational gamma-carboxylation of many proteins, including matrix Gla protein (MGP), which in turn inhibits calcification. Nigwekar et al. could demonstrate a significant decrease of carboxylated MGP in patients suffering from calciphylaxis compared to controls [22].

Another instance demonstrating the relationship between VKA and calciphylaxis was seen in one of our patients surviving calciphylaxis. Initial VKA treatment was begun due to atrial fibrillation. Upon diagnosis of calciphylaxis VKA treatment was stopped and calciphylaxis associated lesions healed completely. After 1 year VKA treatment was continued, however, this lead to a recurrence of calciphylaxis associated lesions. In summary, VKA use in calciphylaxis patients should be avoided, alternatively new oral anticoagulants should be considered as treatment options.

Impaired liver function seems to be, as up to now, an underestimated contributing factor. In our cohort, low serum albumin levels as well as reduced PCHE levels could be identified as contributing factors.

Furthermore obesity seems to be a major contributing factor. None of our patients presented with a normal BMI, the majority had a BMI of > 35 kg/m^2^. However, larger patient numbers are needed to substantiate the connection between obesity and calciphylaxis. A pathogenic factor in obese patients might be due to an increaseed tumor-necrosis-factor-alpha expression in adipose tissue, which in turn decreases blood circulation thus accelerating the calcification process.

Limitations: The analysis of patient charts by a non-blinded observer may be a source of bias. Retrospective evaluation of unstandardised data also poses a limitation to this study. Future studies with larger study populations and a group of controls will be of great interest in order to draw definite conclusions on risk factors for calciphylaxis.

## Conclusion

Calciphylaxis is a rare disease with high mortality associated with female sex, obesity, severe chronic kidney disease and hyperparathyroidism. Especially treatment with VKAs and liver dysfunction are important factors associated with the development of calciphylaxis. Cumulation of multiple concomitant factors seems to lead to disease manifestation. Normal renal function with simultaneous disturbed calcium and phosphate homeostasis in conjunction with secondary hyperparathyroidism, VKA administration or vitamin D therapy can also lead to outbreak of calciphylaxis. In summary, there seems to be no single determinant causing calciphylaxis, however, interaction of different contributing factors with different weight may lead to disease outbreak. Once a certain threshold is exceeded, calciphylaxis breaks out.

All in all it is a life-threatening event for patients.. As a result of our study, physicians should consider the use of DOACs in patients with chronic kidney disease, where possible. As progression and development of calciphylaxis are chronic rather than acute processes, early use of DOACs instead of VKA might be beneficial and reduce the incidence of calciphylaxis. Certain DOACs have been approved for use in patients with moderate to severe and severe chronic kidney disease lately. Unfortunately, data on safety of DOACs in patients with advanced CKD are limited, and studies investigating the risks and benefits of their use in patients at risk for calciphylaxis are direly needed. At the latest, in patients with additional skin lesions and hyperparathyroidism, a switch from VKA to either DOACs or Heparin is highly warranted.

Due to growing awareness of this disease, the incidence of diagnosed calciphylaxis will increase over the coming years. But probably with the recently increasing role of DOACs replacing VKAs, we might see a future decline in the overall incidence of this severe disease.

Further prospective studies investigating these identified factors are required and in part already in progress.

Prospective registries are:

-Sagar Nigwekar (MD): Partners Calciphylaxis Biobank (PCB); (ClinicalTrials.gov Identifier: NCT03032835; available from https://clinicaltrials.gov/ct2/show/NCT03032835?cond=calciphylaxis&rank=8; cited 2019 February 10).

-Vincent Brandenburg (MD): European Calciphylaxis Registry Network (EuCalNet); (ClinicalTrials.gov Identifier: NCT02635373; available from https://clinicaltrials.gov/ct2/show/NCT02635373?cond=calciphylaxis&rank=5; cited 2019 February 10).

Currently ongoing prospective clinical trials:

-Sagar Nigwekar (MD): Evaluation of Vitamin K Supplementation for Calcific Uremic Arteriolopathy (VitK-CUA); (ClinicalTrials.gov Identifier: NCT02278692; available from https://clinicaltrials.gov/ct2/show/NCT02278692?cond=calciphylaxis&rank=9; cited 2019 February 10).

-Craig Sherman (MD): A Phase 3 Clinical Trial of Intravenous Sodium Thiosulfate in Acute Calciphylaxis Patients (CALISTA); (ClinicalTrials.gov Identifier: NCT03150420; available from https://clinicaltrials.gov/ct2/show/NCT03150420?cond=calciphylaxis&rank=3; cited 2019 February 10).

-Jürg Hafner (MD): Pathophysiology of Martorell Hypertensive Ischemic Leg Ulcer (HYTILU); (ClinicalTrials.gov Identifier: NCT01578382; available from https://clinicaltrials.gov/ct2/show/NCT01578382?cond=calciphylaxis&rank=10; cited 2019 February 10).

## Abbreviations

BMI: Body-Mass-Index
CKD: Chronic kidney disease
CUA: Calcific uremic arteriolopathy
DOACs: Direct oral anticoagulants
ESDR: End-stage renal disease
ICD: International Classification of Diseases
MGP: Matrix Gla protein
PCHE: Pseudocholinesterase
PPI: Proton pump inhibitors
PTH: Parathyroid hormone
VKA: Vitamin K antagonists
